# Anent the theoretical justification of a sex doula program

**DOI:** 10.1007/s11017-023-09612-8

**Published:** 2023-02-24

**Authors:** Steven J. Firth, Ivars Neiders

**Affiliations:** 1 University of Helsinki, Helsinki, Finland; 2 University of Latvia, Riga, Latvia

**Keywords:** Sexual citizenship, Disability, Sexual rights of disabled, Liberal utilitarianism, Healthcare rights, Capabilities approach, Sex doula

## Abstract

The Human Condition is neither a well-defined nor well-described concept—nevertheless, it is generally agreed that human sexuality is a fundamental and constituent part of it. For most able-bodied persons, accessing and expressing one's sexuality is a (relatively) trouble-free process. However, many disabled persons experience difficulty in accessing their sexuality, while others experience such significant barriers that they are often precluded from sexual citizenship altogether. Recognising the barriers to the sexual citizenship of disabled persons, the concept of a Welfare-Funded Sex Doula Program has been advanced — a program specifically aimed at meeting the various (and often complex) sexual needs of disabled people. Below we show how that program can be justified within at least two different moral frameworks, the capabilities approach and liberal utilitarianism, and consider and repudiate arguments against it.

## Introduction—the sexual citizenship of disabled persons

It is generally understood that a ‘life worth living’ should contain not only the basic fundamentals of life such as survival and physical health, but should also include things such as: freedom from persecution, love and sex, liberty, and self-determination. Indeed, it is this very belief that has motivated the development of welfare systems from which many now benefit. These fundamental or basic needs are considered a baseline for the quality of life a government owes to its citizens.

The idea that sexual fulfilment is a basic human need is supported in the literature with authors such as John Danaher observing “that having access to meaningful sexual experiences is an important part of the good life,” [1, p. 467] and Jacob Appel arguing that “sexual pleasure is a fundamental human right” [2, p. 153]. Rights-talk aside, we hold that sexual citizenship is fundamental to an individual’s well-being [3] and should be afforded to *all* members of a society [4, 5]. We believe that those precluded from sexual citizenship (by which we mean a sexual agent’s access to, and appropriate participation in, a sexual society) may feel removed or distanced from humanity [6, 7]. Not surprisingly, then, the effects of such exclusion are profound: isolation, social devaluation, reduced self-worth, and depression.

Disabled persons constitute a varied and diverse group of people. Though some disabled people experience full sexual lives, many have difficulty accessing the social environments which facilitate sexual expression and opportunities, and relationship building [8, 9].  Sexual exclusion can obtain in ways that are importantly *not* mechanisms of exclusion for non-disabled persons; [7] that is, as a function of social attitudes around body-image and attractiveness which “may hinder the sexual expression of disabled people;” [6, p. 66] as a function of physical or mental impairments; and perhaps most perniciously, because of the presumption of ‘sexlessness’ [10, 11]. Together with the nature and severity of an individual’s impairments, this presumption has resulted in many disabled people being “denied sex or conversations about sex, sexual expression, and pleasure” [7, 9, 12, p. 363]. Such dispositions exist in the ‘no sex’ policies of nursing homes and can even extend to the parents of disabled persons (who may have difficulty recognising their child as a sexual agent) [7, 9]. This lack of inclusion and communication impedes an individual’s sexual awakening and can compound an already turbulent period of growth.

A disabled person’s parents and caretakers are often their only interfaces with the world and constitute the only resources available to help them come to terms with puberty, development, and sexual awakening [7]. If such conversations are denied, then disabled persons are left with little-to-no ability to frame their sexual development; this may, in turn, result in improper attitudes towards sex and their becoming vulnerable and disadvantaged [7, 12]. Mark O’Brian explains:

No one...had ever discussed sex around me. The attitude I absorbed was not so much that polite people never thought about sex, but that no one did...This code affected me strongly, convincing me that people should emulate the wholesome asexuality of Barbie and Ken, that we should behave as though we had no ‘down there’s’ down there [7].

The addition of vulnerability and disadvantage to sexual exclusion (and its associated psychological trauma) further undermines an individual’s well-being, and many disabled persons find themselves in need of sexual support. Around the world, many non-profit and non-government organisations (NPOs and NGOs), such as the TLC-Trust [13] and Touching Base have stepped up to respond to these needs. These organisations offer varying levels of services from counselling to the provision of sexual relief through masturbation [12]. Some authors, such as Ezio Di Nucci, have argued that charitable responses to the problem are not only sufficient for the needs of disabled persons, but advantageous “as disabled people would probably enjoy it more” [14, p. 160]. We find this position facile [12, 15, 16].

Simply speaking, NPOs are hampered by chronic underfunding, an excessive demand upon their services, and because they are often impeded (ironically) because of barriers to working alongside government disability services. [12] Due to the limited efficacy of charitable organisations a number of alternative responses have been proposed [1, 7, 12, 17] — we believe that the development and state funding of a sex doula program [12] has the most potential [15, 16].

## Sex doulas

Broadly speaking, doulas can be “characterised as non-medical coaches, facilitators, and assistants who offer skilled social, emotional, and practical support” [12, p. 363]. Furthermore, they are trained in skills particularly useful to working with disabled clients such as advocacy, active listening, assisted decision-making, and resource introduction. Their responsibilities are, then, quite different to those of sex counsellors, therapists, or surrogates, who are *generally* understood to focus on sexual disorders and dysfunction. Disabled people's sexual needs are not dysfunctional — they are unattainable. Due to their extended remit, and because of the positive regard in which doulas are held by their clients, the term ‘sex doula’ was coined.

Many different kinds of doulas exist including end-of-life, birth, abortion, and specific-care doulas. ‘Mission creep’ or diversification of existing doula roles is not suggested; instead, sex doulas should receive specific training related to meeting the unique sexual needs of disabled persons. Similar training occurs in Denmark, where interested social workers must complete an eighteen-month long specialised program to become qualified as *seksualvejledere* (sex advisors) [9]. Though the ambit and training of sex doulas has not yet been delineated, it is conceivable that sex doulas would follow a similar training process (in what way such training may differ from the Danish program is not within the scope of this paper).

Danish sex advisors follow a set of recommendations and principles encapsulated in the 1989 *Vejledning vedrørende seksualundervisning og seksualoplæring af mennesker med ophold i institutioner for personer med vidtgående fysiske eller psykiske handicap og i særlige plejehjem* (*Guidance on sex education and sex education for people staying in institutions for people with severe physical or mental disabilities and in special care homes;* hereafter the ‘*Guidelines*’ [4]). This document, developed in conjunction with the personal experiences of disabled persons, explicitly acknowledges that “sexuality is an integrated part of the personality of every person” [9, p. 69] and that disabled individuals “shall have the possibility to experience their own sexuality and have sexual relations with other people" [9, p. 103]. The *Guidelines* engages practical topics such as forming a ‘sexuality plan’ with a client, assisting disabled people to masturbate or to have sex with a partner, and the process of helping procure the services of sex workers [4, 9]. In short, the *Guidelines* constitutes a set of concrete principles surrounding the sexuality of disabled persons in Denmark with an “explicit, articulate attention to sexual pleasure…not well known internationally” [9]. It is our opinion that something similar should (and ought) be developed to underpin programs seeking to advance the sexual citizenship of disabled persons — such as welfare-funded sex doulas.

## Two theoretical frameworks

There exist many theoretical frameworks broad enough to encompass a welfare-funded sex doula program. However, not all frameworks are well-disposed to such a purpose. John Rawls’ *A Theory of Justice*, for instance, sidelines disabled persons — excluding them altogether from the contract situation. More broadly speaking, deontological and natural-law theories can often be sexually conservative^1^ and would, therefore, be resistant to our goal (a short elaboration is made on this matter in the criticisms section). We have selected two robust, well substantiated, and (to some extent) broadly opposing social-justice perspectives: the capabilities approach (CA) and liberal utilitarianism (LU). The former is well represented in the literature while the latter has received less attention — accordingly, we begin our investigation there.

### Just better utilitarianism—liberal utilitarianism

While utilitarian metrics are often incorporated into government cost/benefit analyses (largely for their ease of application), utilitarianism as a theory of justice has been largely dismissed as a viable doctrine due to its internal difficulties [19]. Matti Häyry’s *Liberal Utilitarianism and Applied Ethics*, published in 1994, offers a revisionary and improved species of utilitarianism that is less vulnerable to the traditional objections against utilitarianism [18]. The two core principles of utilitarian theory, the principle of utility and the principle of equality, are preserved — however, LU substantially deviates from classical versions by focusing on “the protection of certain human rights and liberties” [18, p. 88]. According to liberal utilitarianism “it is always wrong to frustrate the basic need satisfaction of beings against their preferences, unless this is the only way to secure the basic need satisfaction of others” [18, p. 126]. The argument by which Häyry arrives at his formulation need not be reproduced here, but the outcome is a minimal ethical theory consisting of the six central normative and axiological principles below.

The first central principle is a transformation of the classical utilitarian principle of utility:

(A) *The greatest need-satisfaction principle*: An act, omission, rule, law, policy, or reform is the right one if and only if it produces, or can be reasonably expected to produce, at least as much need satisfaction as any other alternative which is open to the agent or decision-maker at the time of the choice [18, p. 124].

Häyry notes that different needs may conflict, and situations might require interpersonal trade-offs. A partial solution to this problem is to introduce a distinction between more-basic and less-basic needs:

(B) *The principle of hierarchical needs*: When the need satisfaction produced by various action alternatives is assessed, those needs which are hierarchically at a less basic level shall be considered only if the action alternatives in question do not, or cannot be expected to, produce an effect upon the satisfaction of needs at a more basic level [18, p. 124].

In cases where needs of the same basic level *are* in conflict the traditional solution is to choose that which maximises overall need satisfaction. Of course, this strategy sometimes leads to unappealing conclusions such as the ‘transplant surgeon case’ (where maximising utility can sanction the cutting-up of a passer-by to distribute organs which save the lives of critically-ill patients). Häyry believes that this unpalatable conclusion can be avoided if we admit that utilitarian theorists do not always *have* to choose the maximisation principle. Accordingly, he adds what we shall call the ‘conflict principle:’

(C) *The principle of other-regarding need frustration*: When the need satisfaction produced by various action alternatives is assessed, the most basic needs of one individual or group shall be considered only if the satisfaction of those needs does not frustrate the needs of others at the same hierarchical level [18, p. 124].

The resulting hierarchy of needs leaves one with the difficulty of deciding which needs are more basic than others. Häyry’s response is to adapt Georg Henrik von Wright’s *necessary ends* (such as survival, health, well-being, and happiness) and *contingent ends* (such as new cars, tickets to concerts, and so forth), into the concepts of more and less-basic needs. [18] This distinction results in the fourth axiological maxim:

(D) *The principle of necessary and contingent ends*: Needs are hierarchically at a more basic level if and only if their satisfaction is conceptually linked with the achievement of necessary ends like survival, health, well-being and happiness. Needs are hierarchically at a less basic level if and only if their satisfaction is conceptually linked only with the achievement of contingent ends [18, p. 125].

Häyry now requires a limiting principle that identifies moral subjects, concluding that the capacity to sense a frustration of needs is what makes a being morally relevant:

(E) *The principle of awareness*: When the need satisfaction produced by various action alternatives is assessed, the needs of individual beings shall be considered only if the beings in question can consciously anticipate, sense or perceive, directly or indirectly, the loss of involved in the frustration of those needs [18, p. 125].

Finally, Häyry introduces an anti-paternalistic principle which can be understood as defending the decisions of autonomous decision-makers against contrary (perhaps hegemonic) arguments such as those based on the sanctity-of-life, sexual conservatism, or slippery-slope discourse. This axiom holds that beings capable of autonomy are best positioned to make decisions regarding what they do or do not need:

(F) The principle of autonomy: When the need satisfaction produced by various action alternatives is assessed, need satisfaction which is freely and informedly chosen by autonomous individuals shall be preferred to the need satisfaction of the same individuals which is not [18, p. 125].

So formulated, Häyry's Liberal Utilitarianism shares some vague similarities to Tom Beauchamp and James Childress' Principlism (which in addition to non-maleficience, also includes axioms on justice and the respect for autonomy) [20]. Liberal Utilitarianism, however, surpasses Principlism in comprehensiveness, completeness, and scope. Having now presented, albeit briefly, Häyry’s LU we must move to show how a welfare-funded sex doula program would become justified under such a framework.

#### Liberal utilitarianism and the sex doula program

Liberal utilitarianism measures utility in terms of need-satisfaction, so we must determine whether or not sexual citizenship constitutes a basic need. If it does, then LU would indicate that the sexual needs of disabled persons should be met. One way to do this, is to use the *principle of necessary and contingent ends* to determine whether or not ‘sexual needs’ are appropriately connected to ‘necessary needs.’ LU is unhelpfully abstract here; Häyry mentions such necessary ends as ‘survival’, ‘health’, ‘well-being’, and ‘happiness’ [18, 21] — but falls short of providing either a taxonomy of needs or any sortal by which to derive them.

It falls on us, then, to try and justify our claim that sexual needs are necessary needs. One way to go about this is to note that von Wright’s (and, thus, Häyry’s) necessary and contingent ends framing can be mapped onto David Hume’s three kinds of goods, “the internal satisfaction of our mind, the external advantages of our bodies [and]…the enjoyment of possessions acquired by hard work and good fortune” [22, p. 487]. The first two of Hume’s goods are akin to ‘necessary ends,’ in that they can be concisely represented by the notions of survival and a ‘good life.’ [18] The goods of ‘possession,’ however, constitute what von Wright might call ‘contingent ends’ [18] and Hume claims they are distinct from the other kinds of goods because their privation can be endured “without suffering any loss or alteration.” [22]

A simpler (but less technical) solution, of course, would simply be to suggest that basic needs are those ‘which ensure that we remain in good health’ (implying shelter, food, warmth, intellectual stimulation, and so forth). Given that sexual citizenship can be positively connected to mental and physical health [7, 23, 24], and in understanding that mental and physical health is a well-understood constituent of the good life [6, 7, 9], it thus follows that sexual citizenship must be a constituent of the good life.

### Be all that you can be—a snapshot of a capabilities approach

The capabilities approach originates in Amartya Sen’s work in economics during the 1970s [25–28] – though aspects of it can be traced back to Aristotle, Adam Smith, and Karl Marx [25]. Moving the focus away from standard informational bases such as wealth and income, Sen argues that resources and public goods are of little use if an individual has no real opportunity to apply them [28]. Instead, he claims, what activities one can take part in (what we might call ‘do-ings’) and what one is able to become or make of one's life (what we might call ‘be-ings’) are the substantive freedoms [28]. These be-ings and do-ings can be broadly understood as ‘functionings,’ while ‘capabilities’ refer to the potential combinations of functionings a person may achieve. Accordingly, if a person is to achieve the life they value, they need the ability (what Sen calls ‘opportunity freedom’) and opportunity (what Sen calls ‘process freedom’) to pursue various functioning combinations [28, 29].

In short, the capabilities approach takes ‘means’ and ‘ends,’ and uses them as metrics by which freedoms can be re-framed: thus, societies should focus on ensuring that ‘ends’ (the kinds of opportunities and potentials needed to function) are achievable instead of providing ‘means’ (such as public resources and goods). This focus on real or substantiative freedoms provides a new perspective to determine the threshold conditions of justice and human dignity; thusly the capabilities approach both focusses on comparative quality-of-life (QOL) as well as theorising on justice. Yet comparative QOL calculi are not without their difficulties [30, 31] (Häyry’s response to such difficulties, pre-empting the development of axiom F above, is to prioritise autonomously-chosen QOL decisions [31]).

In the philosophical setting, the capabilities approach is best known through Martha Nussbaum [25, 26, 32–38]. Her formulation has prompted the development of new perspectives surrounding fundamental entitlements and distributive justice, and though philosophical uptake to the CA is becoming more common in disability studies [5, 9, 27, 32–40], the CA has been less employed in relation to the sexual citizenship of disabled persons (with notable exceptions [5, 6, 9, 10, 38, 40]); a lacuna exists on the matter of public policy and disability [36]. If there is an underuse of the CA in applied philosophy, it may be because of measurement and evaluation difficulties, definitional imprecision, a lack of a definitive list of capabilities, and internal difficulties within the framework. An example of the latter can be found in Sen’s resistance to developing a list of capabilities–arguing that it cannot be done without knowing more about the context of each of the capabilities [40].

Nussbaum, however, *has* formulated a tentative list of core capabilities (or fundamental entitlements), which may explain why her formulation has achieved more traction than Sen’s. Her list includes: (1) *Life;* (2) *Bodily Health;* (3) *Bodily Integrity;* (4) *Senses, Imagination, and Thought;* (5) *Emotions;* (6) *Practical Reason;* (7) *Affiliation;* (8) *Other Species;* (9) *Play;* and (10) *Control (over one’s environment)—*“opportunities for sexual satisfaction” are specifically detailed under (3) *Bodily Integrity* [25]*.* Her approach, she claims, is “fully universal,” “extends to each and every citizen,” and is “cross cultural and against the positions of cultural relativists” [25, p. 76]. Nussbaum holds that a society is just when it ensures that its people have enough of the core capabilities that they are able to live a life of human dignity [25]. However, because her formulation stipulates that the core-capabilities are “open-ended and subject to ongoing revision and rethinking” [25, p. 78] the baseline of capabilities could be argued to be in flux. It is, therefore, not altogether clear that a society could ever reach an ‘ideal-state’ of justness.

At its heart, the CA seeks to determine (and perhaps define) a sort of baseline above which people live a life of human dignity and below which people do not. A just society, Nussbaum claims, would not provide its people with an abundance of some capabilities but a lack or absence of others. This instance in non-fungibility likely comes from Nussbaum's being influenced by Rawls’ *Theory of Justice* and the criticism of utilitarian trade-offs to ensure the greater aggregate of happiness made therein. Such criticisms find no purchase in LU due to Häyry’s ‘principle of hierarchical needs’ and the ‘principle of necessary and contingent ends’ – satisfaction of the latter, ironically, could be said to be the fundamental goal of the capabilities approach itself.

#### Capabilities approach and the sex doula program

The extent to which all persons are able to reach a baseline of capability is unclear. Nussbaum claims, for instance, that certain severely mentally impaired persons may never have the internal capacity to attain the capability of control or practical reason. The matter of autonomy for such persons is a complex one — especially when it comes to the matter of sexual preferences and consent [5]. Simo Vehmas notes that legal scholars advocating the UN Convention on the Rights of Persons with Disabilities maintain that “all human persons, regardless of their decision-making capabilities, should enjoy ‘legal capacity’ on an equal basis” [5, p. 528]. He concludes that systems of support (such as supported or facilitated decision making) must be adopted to help determine the person’s decisions — even in those areas which make us ‘uncomfortable’ [5]. This is not to suggest that Nussbaum sidelines persons with mental impairments as moral patients (as does Immanuel Kant) nor are they separated from the contracting group (as they are in Rawls) — her approach is, in part, a direct response to those problems. What she claims is that there is a limit to what capabilities are open to certain individuals in virtue of their uniqueness.

For our purposes, we assume that the disabled persons in question have the innate capacity to be sexual beings (being a sexual citizen is not necessarily dependent upon one’s mental faculties — see [5]). The onus under the CA would then be to ensure that those persons who have the innate capacity for a capability are also able to *function* with respect to that capability — that is, that a person is able to “convert resources (or commodities) into individual functionings” [36]. These ‘conversion factors’ can be ‘internal’ (such as physical conditions, gender, or talents), or ‘external’ (such as one’s environment or social circumstances) [36]. In order for disabled people to reach the baseline of capability and functioning experienced by non-disabled persons, ‘capability inputs’ may be required [36]. Inputs can take the form of changes in societal norms, resources, public policies, infrastructure, etc. [28, 34, 36]. We believe that such inputs can be positive or negative: Negative inputs would make it *more* difficult to convert resources into functionings, such as the recent revisions to the *Guidelines* (which has made it impossible for sex advisors in certain regions to offer to arrange sex worker visits for their residents [9]); while positive capability inputs (such as the provision of a welfare-funded sex doula program) could help disabled persons achieve a baseline of capability and functioning that they may otherwise not. This amelioration is possible because the sex doula program is able to catalyse both internal and external conversion factors: internal conversion factors might include assisting persons to better understand and use their bodies, while external conversion factors may include adjusting the care-home environment to provide welcoming spaces for sexual activities or facilitating social gatherings for care home residents.

More information on exactly how a sex doula program might augment the conversion factors requires further research. However, we can offer an example of how it could be possible to determine where conversion factors need support: Jean-Francois Trani et al. have created a rigorous, semi-structured, survey tool that measures “the gap between one’s performances in terms of functioning and the ideal capability” [36, p. 154]. By modifying their questions to focus on (say) various *aspects* of sexual citizenship (such as a person’s ability to masturbate without support) it would be possible to obtain qualitative and quantitative data regarding an individual’s sexual capabilities. These data would help determine in what way (and to what extent) a person could or could not function; that information, in turn, would then be used by sex doulas to develop an individual’s ‘sexuality plan’ and help them achieve functioning.

Trani et al.’s set of questions are bivalent: PART 1 determines a baseline of functioning, while PART 2 determines the actual (current) level of capability. We are not unaware of the difficulties of communicating with, and determining preferences of, severely mentally impaired persons [5, 9]; and we are mindful that the ‘adaptive preferences’ of disabled persons may skew survey results [37]. Further explication of the modified survey tool goes beyond the scope of this paper, but we maintain that a sex doula program would focus at raising a given capability-aspect with the intention of developing functioning. In as much, we believe that a CA not only supports a welfare-funded sex doula program, but also offers mechanisms by which information can be gathered to direct the support sex doulas should provide.

### Some criticisms: refutation and repudiation

The preceding, we believe, is sufficient to justify a welfare-funded sex doula program under both LU and the CA: Neither LU nor the CA explicitly claims that fulfilment of basic needs (or central capacities) would fall to the welfare state. However, given that the welfare state is that office charged with ensuring that the basic needs of citizens are met, it would be reasonable to assume that such an obligation falls within its bailiwick. We now move to consider some criticisms of a welfare-funded sex doula program.

#### Rights-talk

We are persuaded by Don Kulick and Jens Rydström’s observation that sexual emancipation of disabled persons is often sidelined by the predisposition of academics to continually debate rights-talk [9]; we include this section *only* in an effort to forestall the inevitable rights-based criticisms.

Both LU and the CA employ a conception of rights; though Nussbaum identifies her CA as a species of Human Rights Theory [25], the ‘rights’ therein are politely-couched as ‘fundamental-entitlements;’ and as a contractualist, she must hold that they are to be discussed and contracted-for. Häyry, less coyly, states that “it is, in fact, the protection of certain human rights and liberties that marks the initial deviation of liberal from classical utilitarianism” [18, p. 88]. There is no ‘nonsense upon stilts’ advanced here!

Given the complexity involved with the distribution of benefits and burdens in welfare states, there is reasonable debate over whether or not a disabled person’s rights to sexual citizenship creates a duty on the state to provide a corresponding support service. Such a duty would, however, only follow on the condition that disabled persons have positive claim rights to such services and not simply a ‘licence’ (sometimes called a ‘liberty right’). We submit that LU would generate positive *in rem* claim rights to sexual citizenship, because the principle of *other-regarding needs frustration* is not violated: “it is always wrong,” LU states, “to frustrate the basic need satisfaction of beings against their preferences” [18, p. 126]. Such positive claim rights are not *in personam* (against the person) but are lain, instead, *in cīvītatem* (against the state)*.*^2^ An example of positive claim rights against a welfare state for sexual support for disabled persons can be found in Denmark, where the National Board of Health and Welfare responded by crafting the 1989 *Guidelines* which now inform the training of social workers as *seksualvejledere* [4].

The existence of the above duty leads us to a related concern: whether or not a welfare-funded sex doula program would frustrate the basic rights of sex doulas and other professionals engaged in any such agency. Di Nucci has responded to the sex doula program by raising what he calls his ‘sexual rights puzzle:’

Universal positive sexual rights are incompatible with universal negative sexual rights. If A has a positive sexual right, then that means that there is at least one person who would lack negative sexual rights. Namely the person who would be supposed to fulfil A’s positive sexual rights. If everybody has negative sexual rights, then everybody has the right to refuse to fulfil A’s sexual needs, but then A has no positive right to sexual pleasure [12, 15, 16, 39, p. 1].

Di Nucci’s puzzle, which arises as a function of using confused rights terminology, can be ‘un-puzzled’ as soon as one draws the correct rights distinctions. The rights to sex as they are understood in this paper (i.e., as positive claim rights against the welfare state) do not demand that everyone must be forced to have sex with everyone else — positive sexual claim rights imply only that there is a duty on *the welfare state* to respond to that sexual need (by providing resources, creating programs, etc.). Even if one were to assume that ‘responding to the sexual need’ were to be taken to mean ‘have sex with’ (which it does not), then the preferences of sex surrogates and sex workers would necessarily correspond, *a priori,* with the sexual needs of others.

To elucidate Di Nucci’s mistake, consider a healthcare parallel — *mūnerum medicōrum* (the duties of doctors). Assuming there *are* positive claim rights to healthcare, such rights do not imply that physicians are forced to provide healthcare services or that governments must force physicians to provide such services. Of course, there *are* doctors who perform surgical interventions, and it is their *duty* to do so; yet this duty arises, not through a positive claim right to healthcare, but through a person’s decision to study medicine and become a surgeon. What positive claim rights to healthcare *do* imply is that there is a duty on the welfare state to ensure citizens have access to medical services such as surgery. When Robert Smith (a surgeon at Falkirk and District Royal Infirmary) “undertook two unilateral, above the knee limb amputations, to resolve instances of [Body Identity Integrity Disorder]” [41, p. 81], he performed a surgery that other doctors have refused because it violated their interpretation of *prīmum non nocēre* (first, do no harm). Like the other surgeons, Smith held negative claim rights to not perform such a surgery but waived them, believing instead that the benefits of such a surgery outweighed the harms and that there was a duty on the healthcare system to provide those surgeries (as there was legitimate need). Similarly, positive claim rights to sexual citizenship do not violate the negative claim rights of those who have elected to care for disabled persons’ sexual needs,* quoniam volentī non fit injūria* (since those who consent are not harmed).

#### Feminist concerns

The concept of the sex doula program was first introduced at the European Society for Philosophy of Medicine and Healthcare conference in 2018. One feminist worry extended there was that such a program would reinforce patriarchal gender relations and the idea that men have a *de facto* claim right to the sexual use of a woman’s body. There are two peculiar and implicit presumptions behind this objection: (1) that doulas can only be female and (2) that only male disabled persons seek sexual citizenship. We shall dismiss (2) because it is obviously false and move to (1). Though the etymology of the word ‘doula’ delineated a female slave, contemporary doulas are of all genders. While it is true that birthing doulas *tend* to be female, both the Childbirth and Postpartum Professional Association (CAPPA) and DONA International have trained male birthing doulas [42]; other doulas, such as end-of-life doulas, demonstrate a more even gender distribution.

Yet the above answer, as Danaher observes, would be to offer a glib response to a serious and pernicious issue [1, p. 20]. Recognising that “at least some [sexual inclusion] rights seems to be, *prima facie*, plausible and morally compelling” [1, p. 24]; he reiterates Amia Srinivasan’s worries that “repoliticising desire will encourage a discourse of sexual entitlement. Talk of people who are unjustly sexually marginalised or excluded can pave the way to the thought that these people have a right to sex, a right that is being violated by those who refuse to have sex with them” [1, p. 24]. Srinivasan concludes that sexual experiences are not a distributive good but *sui generis* [43]*.* Such a proposal may seem persuasive, but such experiences must still be *generis — *that is, of a kind that is debatable (and, in our case, enactable). We are sensitive to Srinivasan’s concerns (especially in light of increasing ‘incel’ community numbers), but believe that simply redacting ‘sexual experiences’ from the list of distributive goods is ‘throwing the baby out with the bath water.’ Once the rights are construed correctly (as we have endeavoured to do above), then the concerns to which Srinivasan alludes seem to dissipate — at least so far as the sex doula program is concerned.

Danaher concludes that ‘Misogyny Objections’ are not enough to undermine the project of greater sexual inclusion, but they do demonstrate how the debate is “fraught with risk and that, if done wrongly, could serve to reinforce a discriminatory and oppressive regime” [1, p. 25]. The answer to the problem, he claims is to “build in significant anti-misogyny safeguards to how the project of sexual inclusion is pursued” [1, p. 24]. We agree with this analysis, but add two points: Firstly, the provision of sexual services for disabled persons is* not* the same as advancing sexual inclusion rights for all persons (the inclusion of the former is unlikely to result in any misogynistic norm enforcement). Secondly, disabled persons are themselves a vulnerable, oppressed, and discriminated minority — half of whom are women hoping that the provision of sexual support services will help them obtain sexual citizenship, too! This latter point highlights that the rights which are being advanced are not so much ‘male-claim rights’ as they are just ‘rights.’

#### Auxiliary concerns

Some Kantians may argue that the selling of sex-related services would violate the Categorical Imperative (CI) — namely by using ‘humanity’ as a mere means. Such a perspective, however, fails to recognise the rational agency of choosing such a career. While it is a legitimate objection to argue that some sex workers are engaged in their occupation non-consensually, sex doulas would be obligated to train for several years in order for them to practice, and their career would, thusly, be a matter of choice and deliberation. Furthermore, working as a sex doula *could* be understood as mandated by the CI, if it were seen as a profession which develops one’s own talents so that they better those of an *other’s* humanity (in much the same way as surgeons use their own bodies and intellect to better the well-being of another).

Certain theories, such as some natural law theories, may also be resistant to a sex doula program because it could be perceived as going against the human telos as defined by our true nature; similarly, conservative theories might criticise such a program out of fears that its acceptance would result in the fall from decent, upright society into some sort of state-funded Bacchanal cacotopia. However, whatever principles are presented here (and in other works) that promote sexual inclusion with the goal of *eu zen* (a life characterised by quality and completeness)*,* would likely be insufficient to subdue such fundamentalist perspectives. As such, we leave such efforts to those who are engaged in that debate and to our future work.

In terms of economic analysis, it is impossible to avoid the fact that funding a sex doula program would constitute a drain on a state’s coffers and might frustrate some derivative or secondary needs of citizens. This frustration is justified through the LU principle of *hierarchical needs*, which maintains that no secondary needs should be taken into account until all primary or basic needs are satisfied [18]; the CA, for its part, simply holds that any society that provided certain capabilities but which tolerated a lack or absence of others would be unjust [25, 28]. Our response is to admit to such a conflict but note that it does not undermine the *prima facie* case for welfare-funded sex doulas — accordingly, whatever budgetary dilemma obtains as a result becomes a matter of debate for distributive justice.

Some may claim that we have not argued for *enough* sex for disabled persons. This matter is more tricky to resolve, as what constitutes ‘enough’ is difficult to determine. Not only are there significant cultural perspectives (Kulick and Rydström detail that in Sweden sexual activity is disciplined unless it is hidden away — while in Denmark, it is encouraged as part of ensuring a rich and fulfilling life [9]), but what constitutes ‘enough' for an individual person also varies greatly. In order to respond appropriately to such criticism, then, it would be necessary to analyse a lot more data than are currently available. As mentioned above, it is possible that one function of sex doulas would be to conduct surveys which might enable a better quantitative and qualitative understanding of the needs of disabled persons. In light of this thinking, we believe that the program (and thus, ‘some’) should come first, and discussion over ‘how much’ should be determined by an ongoing process of evaluation and discussion.

Finally, we think that is it possible for our interlocutor to claim that the problem of sexual inclusion is simply unsolvable because of the complexities involved and because of the perpetual rights debate that ensues. Danaher discusses a similar objection, which he coins a species of the ‘impossibility/impracticality objection.’ He believes that such an objection fails to “scupper the project of fostering greater sexual inclusion,” [1, p. 25] and we agree. Whether or not something is ultimately resolvable is not an argument against attempting to solve it (ask anyone working on ‘the nature of the good’).

## Conclusion

We have shown above how a sex doula program can be introduced as a part of the greatest need-satisfaction in Häyry’s liberal utilitarianism — and how doing so would help secure a (currently unattainable) “indispensable element of human happiness and well-being” [18] for disabled persons. We have demonstrated how the program can catalyse both internal and external conversion factors leading to an increase of sexual functioning. In addition, we have illustrated how the provision of a sex doula service would neither frustrate the equally-basic needs nor violate the negative claim rights of others. Furthermore, we have shown how positive claim rights are not *in personam*, but *in cīvītatem*—against the state; and through our example of *mūnerum medicōrum,* we have illustrated how positive claim rights to sexual support services do not (and cannot) violate the negative claim rights of doulas working in the program. We have also established how the sex doula program constitutes a societally enactable policy, consistent within the normative protocol of the capabilities approach, that directly responds to the barriers that cause the sexual exclusion of disabled people and which would ensure that disabled persons function in a way that they currently do not.

Responding to criticisms, we concur with Danaher that the broader ‘Misogyny Objection’ demands only that the matter of sexual inclusion must be handled carefully. In addition, we note that concerns over patriarchal rights-claims that generate ‘misogynistic norm enforcement’ are somewhat hyperbolic given that the subject at hand is the sexual citizenship of disabled people. Di Nucci’s ‘sexual rights puzzle’ (which improperly equates the 'positive healthcare right to sexual services’ with the ‘positive right to sex for disabled persons’) has been considered and repudiated.

Finally, we acknowledge that the above discussion does not constitute an in-depth normative analysis of our proposed program – however, we have sought to provide an overview of how the sexual citizenship of disabled persons could be evaluated and in what areas a sex doula program would need to operate in order to ensure baseline functioning. Our work here seeks only to show that a welfare-funded sex doula program is consistent with (at least) two mainline conceptual frameworks of justice, and we believe that it succeeds in that endeavour.

## Footnotes

1 We would like to thank a reviewer for this observation.

2 We are thankful to a reviewer of this paper for pointing out that no latin term for a positive claim right against a welfare state exists. In an effort to develop useful terminology we would like to propose ‘*in cīvītatem,’* which translates as ‘against the state.’
